# Automating Responses to Patient Portal Messages Using Generative AI

**DOI:** 10.1055/a-2565-9155

**Published:** 2025-07-30

**Authors:** Amarpreet Kaur, Alexander Budko, Katrina Liu, Eric Eaton, Bryan D. Steitz, Kevin B. Johnson

**Affiliations:** 1 Department of Biostatistics, Epidemiology, and Informatics, Perelman School of Medicine, University of Pennsylvania, Philadelphia, Pennsylvania; 2 School of Engineering and Applied Science, University of Pennsylvania, Philadelphia, Pennsylvania; 3 Vanderbilt University Medical Center, Vanderbilt University, Nashville, Tennessee

**Keywords:** patient web portal, physician–patient interaction, artificial intelligence, communication, health, medical informatics, large language models

## Abstract

**Background:**

Patient portals bridge patient and provider communications but exacerbate physician and nursing burnout. Large language models (LLMs) can generate message responses that are viewed favorably by health care professionals/providers (HCPs); however, these studies have not included diverse message types or new prompt-engineering strategies.

**Objectives:**

Our goal is to investigate and compare the quality and precision of GPT-generated message responses versus real doctor responses across the spectrum of message types within a patient portal.

**Methods:**

We used prompt engineering techniques to craft synthetic provider responses tailored to adult primary care patients. We enrolled a sample of primary care providers in a cross-sectional study to compare authentic with synthetic patient portal message responses generated by GPT-3.5-turbo, July 2023 version (GPT). The survey assessed each response's empathy, relevance, medical accuracy, and readability on a scale from 0 to 5. Respondents were asked to identify responses that were GPT-generated versus provider-generated. Mean scores for all metrics were computed for subsequent analysis.

**Results:**

A total of 49 HCPs participated in the survey (59% completion rate), comprising 16 physicians and 32 advanced practice providers (APPs). In comparison to responses generated by real doctors, GPT-generated responses scored statistically significantly higher than doctors in two of the four parameters: empathy ( *p* < 0.05) and readability ( *p* < 0.05). However, no statistically significant difference was observed for relevance and accuracy ( *p* > 0.05). Although readability scores were significantly different, the absolute difference was small, and the clinical significance of this finding remains uncertain.

**Conclusion:**

Our findings affirm the potential of GPT-generated message responses to achieve comparable levels of empathy, relevance, and readability to those found in typical responses crafted by HCPs. Additional studies should be done within provider workflows and with careful evaluation of patient attitudes and concerns related to the ethics as well as the quality of generated responses in all settings.

## Background and Significance

Patient portals have become an integral and indispensable component of modern health care, providing patients with secure online access to vital health information and facilitating crucial communication bridges between health care professionals/providers (HCPs) and patients. In doing so, they foster stronger connections between providers and patients and facilitate the delivery of personalized care through effective communication. 1 However, with the rapid growth of patient portals, especially during the COVID-19 pandemic, 2 this convenience has led to an overwhelming surge in the number of in-basket messages that HCPs must manage daily. This growing volume of these in-basket messages, many of which are administrative or routine, contributes to clinical burnout, reduces efficiency, and diverts valuable time from direct patient care. 1 2 3 4 First documented in 1974, physician burnout has been linked to the demands of EHR documentation, which consumes substantial clinical time. 2 5 Primary care providers face uniquely heightened burnout risks among all HCPs, emphasizing the pressing need for interventions to alleviate EHR-related burdens and support clinician well-being. 2

Generative AI tools (GenAI) such as OpenAI®'s GPT-4 have emerged as promising tools in the health care sector, particularly for mitigating documentation-related burnout among clinicians. GenAI has gained widespread attention in the medical community due to its capacity to effectively generate patient clinic letters, radiology reports, medical notes, discharge summaries, and clinical decision support. 6 7 8 9 10 Researchers have begun to explore the patient-facing role of GenAI in areas such as chatbots, 11 12 wearable technologies 13 14 15 and automating patient portal message responses. 9 16 In particular, a recent study by Tai-Seale et al. with 52 participants showed that GenAI drafts took longer to read, were approximately 18% longer in length, and while responses were less detailed, they were generally more empathetic. Most respondents edited messages to remove recommendations for appointments or inaccuracies. This study did not divide messages into message types, such as those described by Sulieman and colleagues, 17 thereby not addressing if specific types of messages may require less scrutiny or human input. It is critical to note that while GenAI has shown promise in automating routine or administrative messages and easing the load on HCPs, not all patient communications are suitable for automation. Understanding which types of patient messages may require less scrutiny or human input is critical to ensuring both efficiency and safety in health care delivery.

Currently, there is limited research and guidance on how to triage patient messages for potential automation, creating an essential gap in knowledge that can affect the effective integration of artificial intelligence (AI) tools. This study aims to address this gap by evaluating the appropriateness of automating responses to specific message types while also exploring HCP acceptability of GenAI in daily clinical practices. By identifying which messages can be safely handled by AI versus those requiring human attention, this research seeks to optimize health care delivery by streamlining communication workflows. The ultimate goal is to alleviate clinician workload, improve response times for patients, and ensure a high-quality personalized care remains a priority, thus offering a practical solution to a growing issue in modern health care.

## Methods

### Study Setting

This cross-sectional, single-group study was conducted at a major urban integrated academic medical center in the Northeast. We primarily focused on physicians and advanced practice providers (APPs) in internal medicine and family medicine. HCPs in this setting are located in over 50 locations and function as a patient-centered medical home for millions of patients. The study was deemed non-human participant research by the University of Pennsylvania Institutional Review Board. Answering the survey served as implied consent to participate in the project.

### Initial Data Collection and Creation of Synthetic Patient Portal Messages

Considering the sensitive nature of real patient portal messages, we first retrieved a set of 85 patient portal message–response pairs from a repository at Vanderbilt University Medical Center. This set of 85 contained messages from both the provider and the patient, resulting in 170 total messages. All identifying language was removed from each message, with identifying nouns replaced with semantically similar language, allowing us to use them on commercial cloud platforms. No changes were made in tone, urgency, or vernacularity. Two investigators (B.D.S. and K.B.J.) then independently categorized each patient message into the categories as described by Sulieman et al. 17 (management, interventions, problems, referrals, test results, and clinical intervention preparation). Interrater agreement for the categorization of these messages was nearly perfect (kappa = 0.94).

**Fig. 1:**
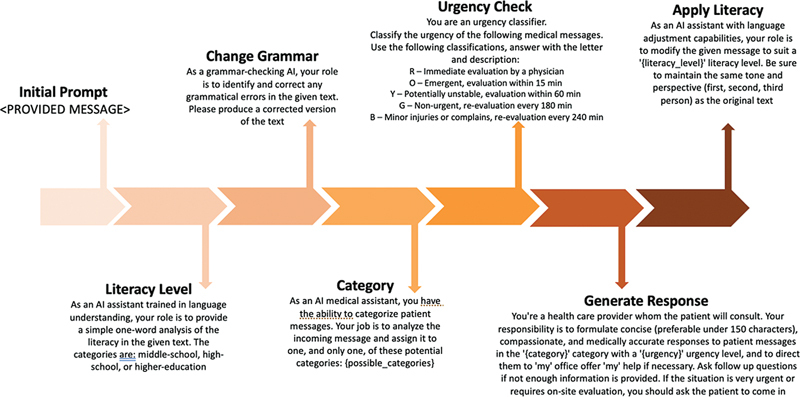
The patient portal message response pipeline using the GPT AI and chain-of-thought prompt engineering. AI, artificial intelligence.

Using the authentic provider responses to these rephrased patient messages, we engineered a prompt within GPT-3.5-turbo, July 2023 version (GPT) 18 to generate additional message responses similar in tone, length, and word choice. We used a strategy based on “chain-of-thought” reasoning, which facilitated the incorporation of explanatory iterative changes in text generated by large language models (LLMs). 19 20 In our use case, we used this strategy to tailor an initial GPT response to the tone and language of the original message. For example, we can modify the response length of a message without compromising accuracy by iteratively modifying a component of the prompt pipeline, as shown in Fig. 1 . A convenience sample of eight experienced primary care physicians reviewed these GPT-generated messages and were unable to distinguish them from the original patient messages.

Given these results, we combined our pool of messages into one set to develop our synthetic patient portal message responses for the second phase of the study.

### Pipeline Development

We used iterative prompt engineering, similar to chain of thought, to create tailored provider responses to patient messages. These engineering prompts contained explicit instructions to mimic the tone, word choices (slang instead of medical words), and brevity of each rephrased patient message but did not include any content from the patient messages used in the test set for GPT-generated responses. Once we were satisfied with the face validity of responses, we generated synthetic patient portal message responses across the range of categories described above, using this prompt:

“ *You're a health care provider whom the patient will consult. Your responsibility is to formulate concise (preferably under 150 characters), compassionate, and medically accurate responses to patient messages in the ‘{category}’ category with an ‘{urgency}’ urgency level, and to direct them to ‘my’ office, offer ‘my’ help if necessary. Ask follow-up questions if not enough information is provided. If the situation is very urgent or requires on-site evaluation, you should ask the patient to come in* .”

The final pipeline is summarized in Fig. 1 , and all codes to generate the messages are available on Github. 21

### Evaluation of Message–Response Pairs

To evaluate the quality and authenticity of messages generated by our pipeline, we conducted a cross-sectional survey study of HCPs across the University of Pennsylvania. The survey consisted of 20 questions, each including a message–response pair. Participants were presented with a total of 20 unique patient messages, with each message accompanied by a provider response. Of these 20 unique patient message–provider response pairs, 10 responses were generated de novo using GPT, while the remaining 10 were written by real doctors. Participants were not informed which responses were generated by GPT and which were provided by real doctors, ensuring that their evaluations were based solely on the quality of the responses, independent of their source. For each question, participants were asked to rate the message–response pairs according to four key quality dimensions of communication, shown by Ayers and colleagues. 22 These quality dimensions aimed to predict the perceived quality of the response: *Empathy* , reflecting the degree of consideration for the patient's emotions in the response; *Relevance* , assessing how closely the response addressed the patient's expressed needs; *Medical Accuracy* , gauging the alignment of the response with established medical practices and guidelines; and *Readability* , evaluating the clarity, coherence, and simplicity of the language employed. Each quality dimension was presented as a Likert-scale question with five possible responses, ranging from 0 to 5. Additionally, participants were asked to discern whether each message response was GPT-generated or written by a real provider.

We recruited HCPs who identified as primary care MDs, DOs, or APPs through an email distribution list to complete the survey. This sampling frame covered most primary care providers at our institution. Eighty-four potential participants responded to the initial email request, and a survey link was sent to each interested participant. Upon completion of the survey, participants received a $10 Starbucks gift card as a token of appreciation. The survey was distributed using both Google Forms and REDCap, the latter being utilized due to firewall restrictions preventing Google Forms use on some computers. The survey was administered between November 28, 2023, and January 5, 2024. We used JMP (version 17.2.0) 17 to complete univariate analyses and to determine if participants who could discern which messages were generated by GPT differed in their assessment of message quality.

## Results

Of the 84 HCPs who showed interest in participating in the survey, 49 completed the survey, resulting in a 58% completion rate. Table 1 provides an overview of various demographic and professional variables among the 49 respondents. Most participants identified as female (77.6%), with 69% between the ages of 31 and 40. A total of 67% of respondents identified as APPs, while 33% held a medical degree (MD or DO). Years of experience seeing patients varied, with the largest group having less than 5 years of experience (31%), followed by experience between 10 and 15 years (18%). Most respondents worked in clinics (69%), in urban settings (63%), and reported receiving 25 to 75 in-basket messages from patients during a typical work week (55%). Most respondents (76%) indicated no or unknown experience with AI tools in medical practice.

**Table 1:** Overview of participant demographics, medical education and specialization, and current medical practices

General demographics	*n* = 49, *n* (%)
Gender	
Male Female	11 (22.4) 38 (77.6)
Age (y)	
25 26–30 31–40 41–50 51–60 >60	0.(0) 5 (10.2) 19 (38.78) 15 (30.61) 5 (10.2) 5 (10.2)
Medical degree	
MD or DO Advanced practice provider	16 (32.65) 33 (67.35)
Years of experience seeing patients	
<5 5–10 10–15 15–20 20–25 25–30 30–35 >35	15 (30.61) 7 (14.29) 9 (18.37) 5 (10.2) 5 (10.2) 2 (4.08) 4 (8.16) 2 (4.08)
Clinical setting	
Hospital Clinic Private setting—Solo practice Private setting—Group Practice with 1–5 providers Private Setting—Group Practice with >5 providers Outpatient specialty practice on hospital campus Long-term care/Office split Other	2 (4.08) 34 (69.39) 0.(0) 3 (6.1) 6 (12.24) 1 (2.04) 1 (2.04) 2 (4.08)
Geographic location	
Urban Suburban Rural	31 (63.27) 18 (36.73) 0 (0)
Number of patients seen during work week	
<20 20–40 40–60 60–80 80–100 >100	6 (12.24) 16 (32.65) 11 (22.45) 11 (22.45) 4 (8.16) 1 (2.04)
Number of in-basket messages received from patients during the work week	
<25 26–50 51–75 76–100 101–200 >200	10 (20.41) 15 (30.61) 12 (24.49) 5 (10.2) 7 (14.29) 0.(0)
Experience with AI tools in medical practice	
Yes No Not sure	4 (8.16) 37 (75.51) 8 (16.33)

Abbreviation: AI, artificial intelligence.

**Fig. 2:**
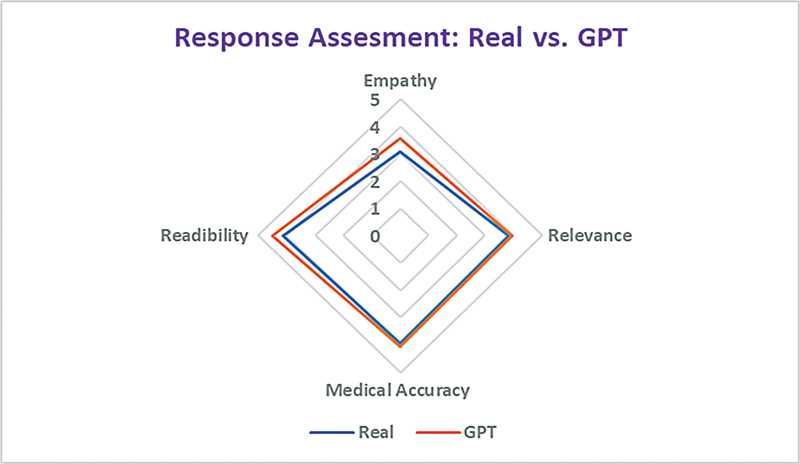
Evaluation of the pipeline. The radar diagram illustrates the mean comparison of GPT-generated and real responses using an ordinal scale ranging from 1 (low) to 5 (high). A rating of 1 indicates poor performance, while 5 signifies excellent performance.

Table 2 and Fig. 2 summarize the overall assessment of message–response quality. Notably, GPT-generated responses generally outperformed real responses across all key characteristics, demonstrating statistical significance with empathy ( *p* < 0.001) and readability ( *p* < 0.001). Relevance also trended toward significance ( *p* = 0.08). Participants correctly identified GPT messages 73% of the time (good guessers) and correctly identified authentic messages 50% of the time ( Table 3 ). There were no statistically significant differences in assessed message–response quality for good guessers versus other participants as determined by one-way analysis of variance (ANOVA; F[1,47] = 2.27, *p* = 0.13).

**Table 2:** Comparative analysis of GPT versus real message responses

	GPT response		Real response		Significance ( *t* -test)
	Mean (± SD)	Median	Mean (± SD)	Median	*p* -Value
Empathy	3.57 (1.02)	3.6	3.07 (1.00)	3.1	<0.001
Relevance	3.94 (1.00)	4.2	3.81 (1.09)	4	0.08
Medical Accuracy	4.05 (0.92)	4.2	3.95 (0.99)	4	0.12
Readability	4.50 (0.68)	4.9	4.13 (1.01)	4.7	<0.001

Abbreviation: SD, standard deviation.

Note: The table above provides a comprehensive breakdown of the average means and medians derived for the four key characteristics, comparing GPT-generated message–response pairs to real ones. Both empathy and readability were statistically better for GPT-generated responses.

## Discussion

In this study, HCPs evaluated the quality of synthetic (GPT-generated) versus authentic (provider-generated) message responses. The results revealed that responses generated by GPT achieved statistically significant ratings in empathy and readability, with a notable trend toward statistical differences in relevance and medical accuracy compared with authentic patient portal message responses. The AI's capacity to generate empathetic and easily readable messages may stem from the technology's ability to invest unlimited time in crafting responses, as opposed to real doctors, who often face time constraints and stress when generating responses to patient messages. These findings not only build upon but also validate previous research by Ayers and colleagues, 22 where a small team of HCPs rated online chatbot responses as more empathetic than verified physician responses. Through a more tailored approach to prompt engineering, these results complement those of a recent study by Tai-Seale and colleagues, where participants found the technology favorable but suggested less “robotic” responses that required less editing. 16 However, it is important to note that our study did not find significant differences in two critical measures—relevance and accuracy—between AI and provider-generated responses. This indicates that while AI may enhance certain aspects of communication, such as tone and readability, it may not yet be superior to human input in areas requiring nuanced judgment, such as the relevance of the response to a patient's medical issue or the accuracy of clinical information. Moreover, while readability differences were statistically significant, the absolute difference was relatively small, raising questions about the clinical significance of these results. This emphasizes the need for careful interpretation of these findings, particularly when considering the practical impact on patient care.

Our study extends these findings by including more response types and utilizing chain-of-thought reasoning to create more nuanced and explainable output. Another study by Garcia and colleagues 23 assessed AI-generated reply utilization and found an overall utilization rate of 20% across 162 clinicians in primary and specialty care, despite draft replies being available for more than significantly more messages. Utilization was affected by technical limitations or internal exclusion criteria, preventing the generation of a message response. Themes affecting adoption included tone, content relevance, and accuracy. Based on our findings, although the complexity of the prompt engineering to create these messages may be high, that cost may result in improved adoption and is likely to be easier to implement over time.

Additionally, it is critical to recognize that this study was not designed as a non-inferiority trial, meaning we cannot definitively conclude that AI-generated responses are comparable to human responses across all quality parameters. Further research is needed to evaluate whether AI tools can consistently maintain high standards of clinical relevance and safety, especially in more complex patient interactions.

As AI-enabled messaging systems continue to mature and advance, with attention to message tailoring and the specific needs of patients from diverse backgrounds, chatbots and similar tools are likely to become more commonplace in medicine. Already, several studies are exploring the feasibility of integrating systems such as GPT to generate high-quality responses to patient inquiries and aid clinical decision-making across various medical specialties. 7 24 25 While AI has shown promise in alleviating the communication burden on HCPs, its use should be considered a supportive tool rather than a replacement for human expertise. AI may be best suited to handle certain types of patient messages that require less medical nuance but should not yet be fully relied upon for more complex decision-making without human oversight.

Of note, crafting effective prompts in our study entailed iterative trial and error. The potential for performance variation underscores the importance of understanding the model's reliance on training data patterns and ensuring the relevance and quality of examples provided. Our resulting strategy and prompts are available for reference, providing valuable insights for future research and implementation endeavors in this rapidly evolving field.

## Limitations

This study is subject to several limitations that may impact its generalizability. First, the sample size of both generated messages (8) and participating survey respondents (49) is small, potentially limiting the breadth of perspectives represented. All participants were drawn from a single health care system, which may not fully capture the diversity of opinions regarding the value proposition for patient portal message responses or the preferred format and comprehensiveness of these responses across different health care settings. Furthermore, the study relied on a convenience sample of providers who may have had more time and interest to participate in the survey, introducing a potential bias in the results. As such, caution should be exercised when generalizing the findings of this study to broader populations.

The patient portal messages used to generate these synthetic provider responses were de-identified, with identifying nouns replaced. At the time of this study, we were not permitted to use Health Insurance Portability and Accountability Act (HIPAA) safe harbor-compliant messages outside of the health system firewall. We anticipate that health systems will relax this constraint shortly, which will facilitate larger studies within a health system. Finally, our use of prompt engineering to generate responses is currently a trial-and-error process, with features of messages proposed by our research team. Our study was conducted using GPT 3.5-turbo, which is an older and less functional version of GPT than would be used in any clinical trials of this innovation. Our study also tested only the capabilities of GenAI in isolation. Clinical trials of this technology should combine chart content, message threads, and patient preferences, possibly leveraging retrieval-augmented generation or expanding context windows to improve the personalization of responses.

It will be important to better understand the desirable characteristics of patient portal message responses from the perspective of HCPs and patients.

### Potential Biases

The survey was administered to HCPs via Google Forms and REDCap. However, no specific measures were put in place to prevent participants from collaborating or discussing the survey or their responses with one another. As a result, there is a potential for response bias or social desirability bias to have been introduced. HCPs may have been influenced by side conversations or peer expectations, leading to responses that were shaped by group discussions rather than independent, unbiased opinions, which could affect the validity of the survey results.

Furthermore, while the survey aimed to capture a broad perspective from HCPs, we did not employ a purposive sampling approach to specifically target participants with varying levels of experience with AI tools in medical practice. This limitation may have introduced sampling bias and influenced the diversity of responses, as HCPs with more experience using AI might have responded differently compared with those with less or no experience. Future studies could benefit from purposive sampling to ensure a more representative sample in terms of AI familiarity, which may provide deeper insights into the technology's impact on clinical practice.

## Future Work

Considering the limitations of our pipeline, several areas for future research and improvement emerge. Quantitative assessments are crucial to validate the significance of each step in the pipeline, offering empirical evidence to support the theoretical justifications for the architecture's structure. The grammar editing phase requires refinement to prevent overcorrection or unintended alterations of colloquial or non-standard language, thus preserving contextual appropriateness.

Continued exploration and adaptation of the underlying model will be necessary to align with evolving understandings of response coherence and relevance. Addressing biases and inaccuracies originating from the training data is imperative to improve system performance and mitigate potential data-driven biases in generated responses. Enhancing the system's capacity to retain context throughout extended or complex conversations can be challenging and must be monitored. Finally, the rapidly evolving nature of LLMs is likely to improve the ability to tailor responses. Systems employing these tools must be periodically reevaluated and refined to support better alignment with the needs of patients and HCPs. For example, we can anticipate changes in multistep pipelines to do more at each step (using recursive approaches and context expansion) without introducing hallucinations and include validation layers to check for many more aspects of readability, bias, and context specificity. These advances and others have the potential for significant improvements over current methods.

The study had inadequate power to assess the importance of some covariates that might be useful for implementing this functionality at scale, including patient and primary care provider characteristics. It will be critical to ensure their efficacy considering patient preferences, health care settings, and regulatory requirements. Further research should be done to understand these characteristics, to understand and address any potential ethical and liability considerations related to automating message responses, and to assess the need for additional studies, particularly those designed as non-inferiority trials, to explore the potential risks and benefits of integrating AI into patient communication workflows. Considering these limitations, while the pipeline offers a promising approach to generating human-like responses, ongoing research and iterative refinements are crucial to enhance its efficacy and applicability in diverse real-world scenarios. Moreover, assessing AI performance across more diverse and clinically complex message types will be critical in determining its broader applicability in health care. By tackling these difficulties and utilizing advances in AI, health care communication may develop to meet patients' and clinicians' ever-changing requirements and expectations.

## Conclusion

The findings of this study suggest that GPT-generated provider responses using new prompt-engineering approaches are acceptable to primary care providers. The study provides promising insights into the potential of AI-driven messaging systems to alleviate clinician burnout and enhance patient communication. As with all technological endeavors, continual evolution is paramount for addressing challenges and leveraging emerging insights from both the technological and cognitive domains.

## Clinical Relevance Statement

This study describes a strategy to create provider responses to patient portal messages using GPT. HCPs found these generated responses to have a comparable level of accuracy, relevance, empathy, and readability to authentic provider responses.

## Multiple-Choice Questions

1. Which of the following best describes the author's approach to prompt engineering?
  - Asking HCPs to describe how they think about creating a patient response.
  - Developing a response using a component of the original thinking of the patient.
  - Applying a sequence of defined tasks in successive prompts to create a tailored and interpretable response. (correct answer)
  - Creating a prompt that gives the model engineers the flexibility to interpret the user's intention.
  **Correct Answer** : The correct answer is option c. Prompt engineering is defined as the process of designing inputs for AI tools that will produce optimal outputs. In the case of this manuscript, the authors applied an iterative prompt engineering approach, summarized in Fig. 1 .
2. What are the four ways that the authors compared GenAI responses to authentic HCP responses?
  - Relevance, readability, medical accuracy, and empathy (correct answer)
  - Readability, length, timeliness, accuracy
  - Relevance, length, actionability, empathy
  - Relevance, readability, urgency, length
  **Correct Answer** : The correct answer is option a. As is becoming a standard evolution strategy for LLM output in patient care settings, the authors examined the output's relevance, readability, medical accuracy, and empathy with results summarized in Fig. 2 .

**Table 3:** Overview of message–response pairs along with the distribution of how participants identified these pairs

Message–response pair	Message category a	Response type	Percentage identified as GPT ( *n* = 49)	Percentage identified as real ( *n* = 49)
**Message:** Ben is having a few problems. His stomach is extended with possible fluid retention. He took his fluid medicine yesterday but it doesn't seem to have helped just yet. He is also becoming very short of breath when doing anything. I wasn't sure if he could be seen in clinic soon or if he could have some blood work done to check levels. **Response** : I'm really sorry to hear about Ben's symptoms. It's really important to get him checked out as soon as possible. Can you please bring him to the clinic so the doctors can give him a thorough check-up and do the right tests?	Medical management	GPT	36 (73%)	13 (27%)
**Message:** My R elbow is hurting. If I schedule an apt, may I receive another injection? My elbow surgery is scheduled in Oct. **Response** : I'm sorry to hear that your elbow hurts. It would be a good idea to make an appointment to have a doctor check your symptoms and talk about the possibility of getting another shot.	Medical interventions	GPT	33 (67%)	16 (33%)
**Message:** Do I need to prepare in any way or the upcoming fluid deprivation test? such as quit drinking fluids at a certain time or avoid caffeine and alcohol in the time leading up to it? **Response** : The test usually finishes faster if you minimize fluid intake after midnight. However, you do not need to strictly avoid drinking anything after midnight and should not do so if you are noticeably thirsty.	Informational—preparation	REAL	21 (43%)	28 (57%)
**Message:** Good morning, I have been dealing with this crud for 8 days now. I still have this terrible cough that I cannot get rid of and when I breathe it doesn't feel like I am getting any air. It is keeping me up at night. I am coughing up/blowing out of my nose this bright yellow clumpy nasty stuff and I am just really tired of the cough. Any suggestions on what else I can do to help this clear up? **Response** : So sorry to hear you've been dealing with this for such a long time. I'd recommend you come in for an acute visit or go to one of our walk-in clinics if closer to your home. We can be sure there isn't something that needs more than simple over-the-counter treatment. It's difficult to fully assess your symptoms and severity of illness without seeing you in person.	Medical—problems	Real	22 (45%)	27 (55%)
**Message:** I need to talk to someone about possibly being referred to see a therapist. I've began to feel very unlike myself. This happened with my previous pregnancy, and it led me to make some hasty not very well planned decisions. I feel very defeated physically and mentally. If there is someone you can refer me to I'd appreciate it. **Response** : Thank you so much for reaching out. Yes, lets get you to see a counselor so that we can get you feeling more like yourself. We have a counselor that works at our office. She is funded by a grant so your appointments with her would be free and we could schedule them the same day as your visit so that you don't have to make extra trips. Are you open to meeting with her?	Medical—referrals	Real	12 (24%)	37 (76%)
**Message:** Please let me know when you get my results. **Response** : I will inform you once I receive your laboratory results. If you have any worries or inquiries, please don't hesitate to contact me.	Logistical—tests	GPT	41 (84%)	8 (16%)
**Message:** I am feeling much better but still have sinus pressure and congestion. I am not sure if I should start the antibiotics or not. **Response:** I'm happy to hear that you're feeling better. Sometimes, even after the infection is gone, sinus pressure and congestion can still stick around. You could try using decongestants or saline nasal rinses that you can buy without a prescription. If your symptoms get worse or don't go away, please contact our office so we can check it out.	Medical—management	GPT	21 a (44%)	27* (56%)
**Message:** Good morning! It looks like everything is stable and normal. So why do I feel so crummy? Will switching meds help knowing my results are normal? **Response:** Your inflammation markers have not been significant elevated, so we cannot necessarily rely on them to assess your disease. Treatment change is recommended based on your report of increased symptoms of joint stiffness, swelling and improvement after taking steroids.	Medical—management	REAL	29 (59%)	20 (41%)
**Message:** Ben is having a few problems. His stomach is extended with possible fluid retention. He took his fluid medicine yesterday but it doesn't seem to have helped just yet. He is also becoming very short of breath when doing anything. I wasn't sure if he could be seen in clinic soon or if he could have some blood work done to check levels. **Response:** I spoke with the doctor about this. Please continue to monitor for the next 24 hrs. If his condition continues to worsen, he should go to the ER. The doctor will call you this afternoon to check in.	Medical—management	Real	23 (47%)	26 (53%)
**Message:** Have you received results from biopsy from July 19? **Response:** Your biopsy was negative. Please call our office to schedule a consult to discuss your plan of care.	Logistical—tests	Real	36 (73%)	13 (27%)
**Message:** I've had my first physical seizure this morning. It was just a facial muscle twitch and pulse no passing out. **Response:** I'm sorry to hear that you had a seizure. It's really important to figure out why it happened. Let's make an appointment so we can talk more about it and decide what to do next.	Medical—problems	GPT	35 (71%)	14 (29%)
**Message:** I just read the X-ray report from my visit. I'm concerned with the Cardiomegaly. Is the enlarged heart due to the stomach being in my chest? **Response** : Cardiomegaly means that the heart is bigger than it should be, and there can be different reasons for this. It's not likely to be connected to the stomach being in the chest. I suggest talking to your doctor about the results to get more information and to understand what it means.	Medical—tests	GPT	32 (65%)	17 (35%)
**Message:** Jacob's mouth sore is really bothering him to talk and eat, and kinda swollen. He is going to get the mouthwash in the morning. How will we know if he needs to see a doc or do we just treat the symptoms? **Response:** If the sore in your mouth keeps bothering you or gets worse even after using mouthwash, or if you notice any signs of infection like a fever or redness that is spreading, it's a good idea to see a doctor. They can check it out and give you the right treatment.	Medical—problems	GPT	28 (57%)	21 (43%)
**Message:** This thing in my throat is continuing to progress. I now have a steady dull pain along with a few random stabbing pains in that area. Swallowing has become a bigger issue also. I have an appointment with you this Friday, and with speech pathology afterwards. I am ready to get this thing out! **Response:** I tried to call you several times today. I am sorry to have not gotten in touch. I hope that your symptoms do not worsen rapidly. If they do, you can come to ER here at any time day or night and we will take care of you	Medical—problems	REAL	11 (22%)	38 (78%)
**Message:** I have an appointment with [DOCTOR] today at 1 and I will have him talk to Jane too, but I'm worried. Since late November, I have been on a mania-like rollercoaster of anxiety that is affecting my sleep. I'm just worried it's going to start physically affecting me. Is there anything that I can do? **Response:** It would be best to discuss this in the office. My staff will contact you to make an appointment.	Medical—problems	REAL	34	15
**Message:** Can you tell me what type of test this is: Natriuretic Peptide, as it appears that I am well over the range. Thanks. **Response:** The Natriuretic Peptide test is used to measure the levels of a hormone that is released by the heart when it is under stress. If the levels are higher than normal, it could be a sign of heart failure or other heart-related conditions. We kindly request that you schedule an appointment to discuss your test results in more detail.	Informational—tests	GPT	45 (92%)	4 (8%)
**Message:** We have questions re: test results. Please call when possible. **Response:** I'm sorry to hear that you're confused about your test results. Don't worry, I'm here to assist you. Can you please give me more information about the test and what's bothering you?	Medical—tests	GPT	46 (94%)	3 (6%)
**Message:** Good morning. I have noticed since Sunday that I has ptosis on the right side it is not worse but it is not better. I am asymptomatic otherwise, but I get tired easy. What do you think? **Response:** I'm sorry to hear about your ptosis, which is a drooping of the eyelid. It's important to look into this more. I suggest making an appointment with me so we can talk about your symptoms and do a physical exam.	Medical—problems	GPT	42 (86%)	7 (14%)
**Message:** Please let me know when you get my results **Response:** [DOCTOR] believes that this probably due to recent steroids for bronchitis, but you also had some elevated white blood cell count in the past also. It is not uncommon in our obese patient population to have elevated white blood cell (WBC) counts. Typically this elevation is not due to underlying marrow pathology but rather reflects low-grade inflammation. Please have your PCP send us some older CBC results for comparison and establish your normal range?	Medical—tests	Real	24 (49%)	25 (51%)
**Message:** I discovered that I have latent TB from an occupational screening . The exposure was during some construction work years ago but they will not treat me. My daughter is immune compromised due to medications she takes. My concern is that my insurance will not treat latent TB. However if I wait until I am active it is already too late and my family has been exposed. More importantly my daughter who's immune system is suppressed. **Response:** [DOCTOR] would like to meet with you in clinic to discuss in more detail. Are there days and times that work well for you?	Medical—problems	Real	33 (67%)	16 (33%)

a Message category interrater agreement 95% (Cohen's kappa 0.94: near perfect agreement).
